# Supplementary material for: Structural mechanism of voltage-gated sodium channel slow inactivation
Source: Nat Commun. 2024 May 1;15:3691. doi: 10.1038/s41467-024-48125-3 (PMC11063143; doi:10.1038/s41467-024-48125-3)
Supplement: Supplementary file 1 — Supplementary Information [file 41467_2024_48125_MOESM1_ESM.pdf]

Supplementary Information for

Structural mechanism of voltage-gated sodium channel slow inactivation

Authors

Huiwen Chen, Zhanyi Xia, Jie Dong, Bo Huang, Jiangtao Zhang, Feng Zhou, Rui Yan, Yiqiang Shi, Jianke Gong, Juquan Jiang, Zhuo Huang, Daohua Jiang

This file contains Supplementary Figures 1-12 and Tables 1-2.

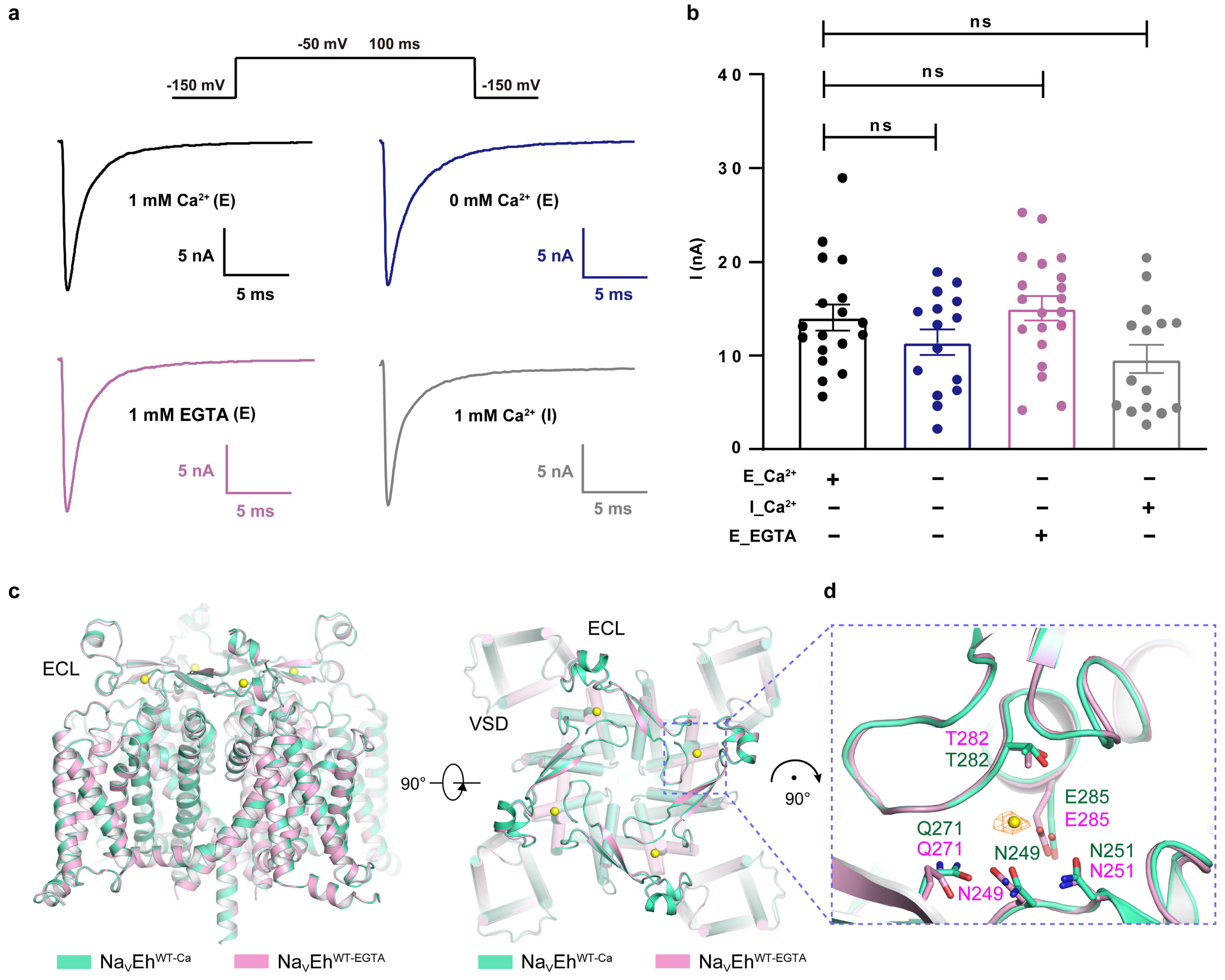

Supplementary Figure 1. Ca^{2+} independent activation of Na_VEh^{WT}

a. Example peak current traces for HEK293T cells expressing Na_VEh^{WT} channels measured at -50 mV upon the addition of Ca^{2+} or EGTA. A schematic diagram of the protocol is presented on the top. E and I represent external solution and internal solution, respectively. **b**. Peak current amplitude of Na_VEh^{WT} in different solutions. External and internal solutions are presented in Supplementary Table 2. Each dot represents a single-cell recording. The removal of Ca^{2+} in the solution has no impact on Na_VEh^{WT} currents. Significances were determined using ordinary one-way ANOVA. P=0.2679 (E_ Ca^{2+}, n=18; E_0 Ca^{2+}, n=15; E_EGTA, n=20; I_Ca^{2+}, n=15). **c.** Structural comparison of $Na_VEh^{WT\_Ca}$ and $Na_VEh^{WT\_EGTA}$. The overall structures of $Na_VEh^{WT\_Ca}$ (green) and $Na_VEh^{WT\_EGTA}$ (light pink) are shown viewed from side (Left) and top-down (Right). The yellow spheres represent putative bound cations. **d**. Zoom-in view the ECLs of $Na_VEh^{WT\_Ca}$ and $Na_VEh^{WT\_EGTA}$. The EM density for putative cation is shown in orange meshes contoured at 12 σ. The surrounding residues are depicted as sticks. Source data are provided as a Source Data file.

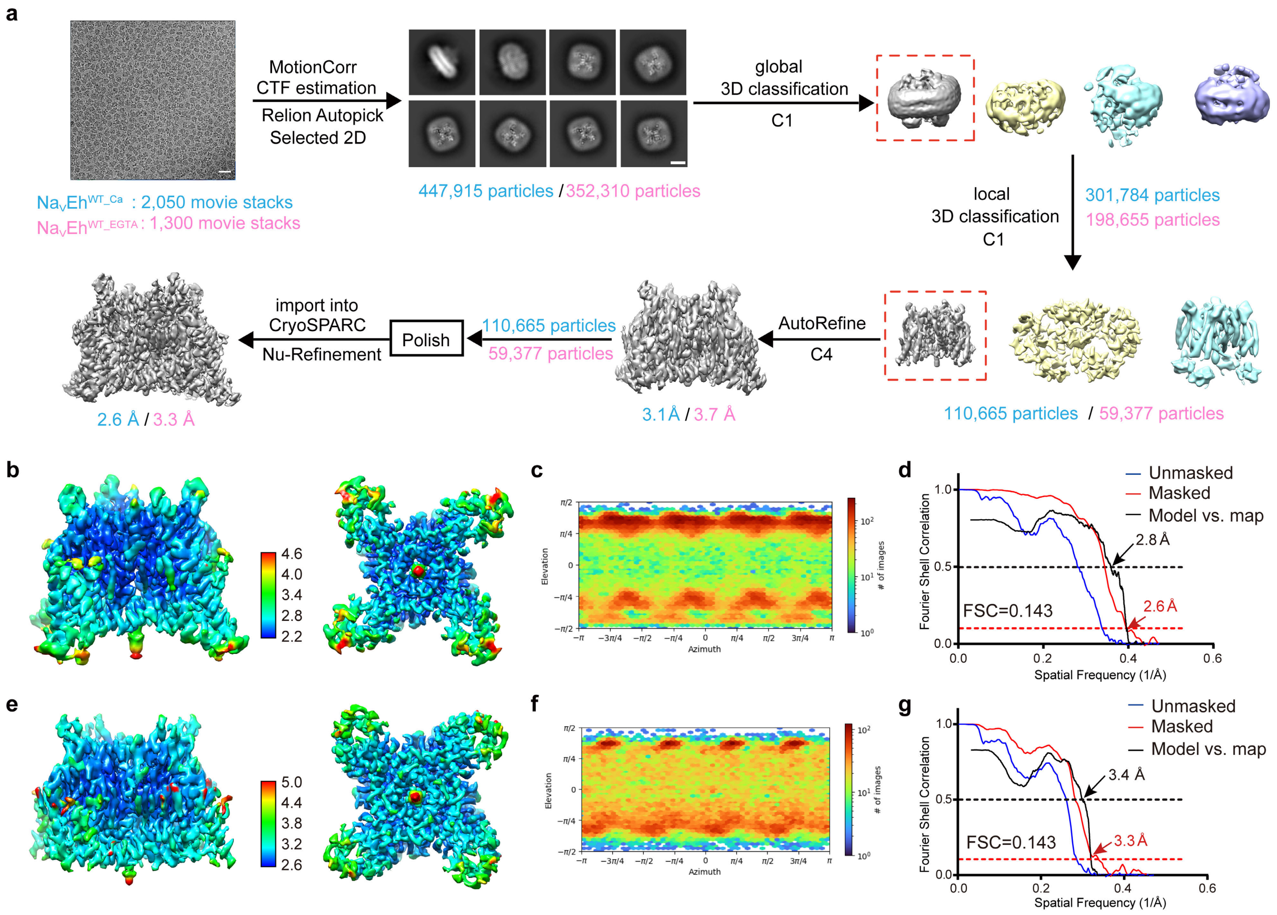

Supplementary Figure 2. Cryo-EM data process of $Na_VEh^{WT\_Ca}$ and $Na_VEh^{WT\_EGTA}$.

a. The flowchart of data process of $Na_VEh^{WT\_Ca}$ and $Na_VEh^{WT\_EGTA}$. Raw movies of $Na_VEh^{WT\_Ca}$ and $Na_VEh^{WT\_EGTA}$ were motion corrected, and the CTF values of the resulting micrographs were estimated by gctf, followed by 2D and 3D classifications. The best 3D class of $Na_VEh^{WT\_Ca}$ and $Na_VEh^{WT\_EGTA}$ contain 110,665 and 59,377 particles, respectively, which were polished in Relion3. And the polished particles were imported into CryoSPARC for non-uniform refinement. **b** and **e**. Local resolution distribution of $Na_VEh^{WT\_Ca}$ (**b**) and $Na_VEh^{WT\_EGTA}$ (**e**). **c** and **f**. Particle angular distribution for the final reconstruction of $Na_VEh^{WT\_Ca}$ (**c**) and $Na_VEh^{WT\_EGTA}$ (**f**). **d** and **g**. The FSC curves for the EM structures of $Na_VEh^{WT\_Ca}$ (**d**) and $Na_VEh^{WT\_EGTA}$ (**g**).

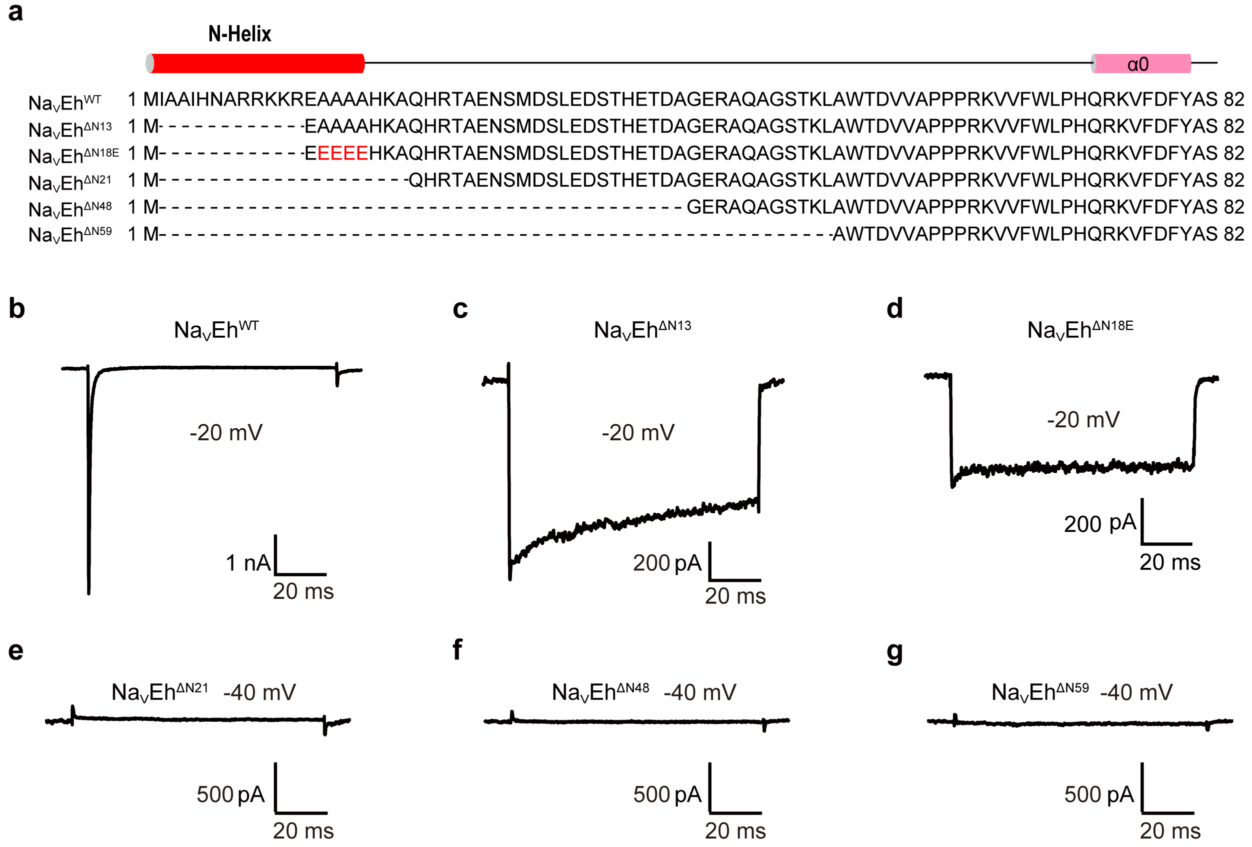

Supplementary Figure 3. Example current traces of Na_VEh mutants.

a, The Na_VEh mutants with varied N-terminal truncations. **b-g,** Example current traces of Na_VEh^{WT} (**b**), $Na_VEh^{\Delta N13}$ (**c**), $Na_VEh^{\Delta N18E}$ (**d**), $Na_VEh^{\Delta N21}$ (**e**), $Na_VEh^{\Delta N48}$ (**f**), $Na_VEh^{\Delta N59}$ (**g**).

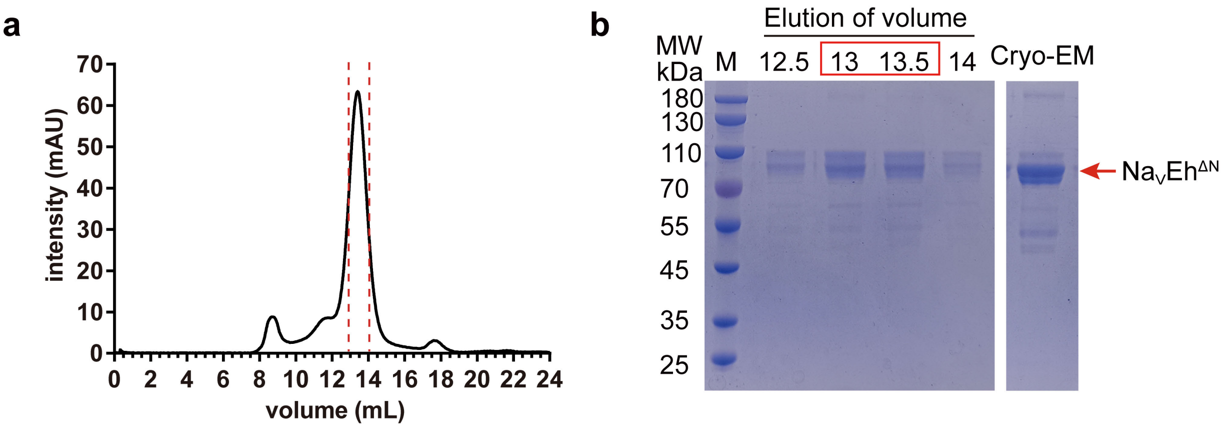

Supplementary Figure 4. Purification of $Na_VEh^{\Delta N}$.

a. A representative size exclusion chromatogram profile of the purified $Na_VEh^{\Delta N}$. Peak fractions between red dashed lines were collected and concentrated for cryo-EM study. **b.** The samples of peak fractions were stained with Coomassie brilliant blue on SDS-PAGE gel. The red arrow indicated the band of $Na_VEh^{\Delta N}$. The SEC profile and gel image are representative of 3 experimental replicates. Source data are provided as a Source Data file.

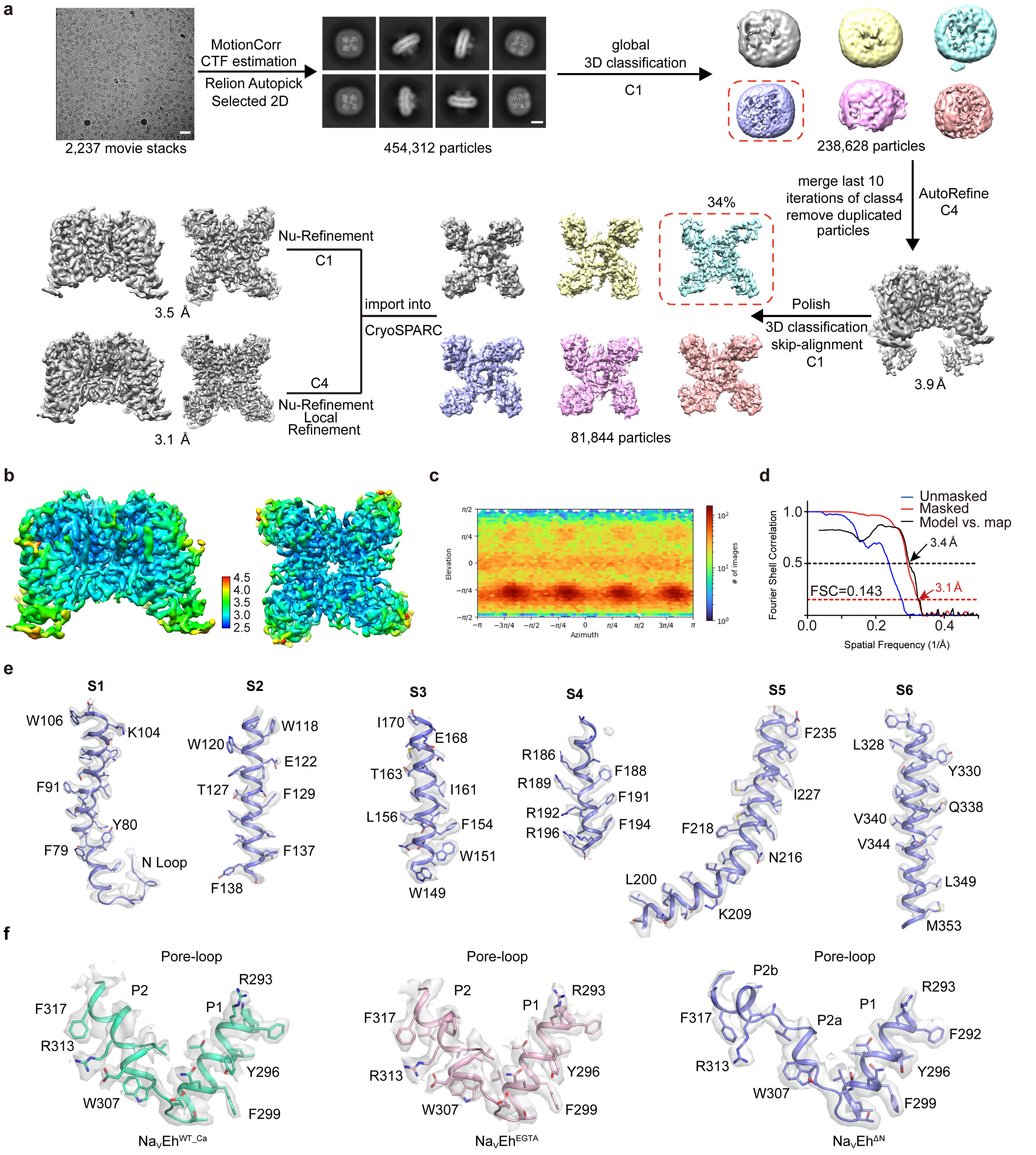

Supplementary Figure 5. Cryo-EM data process of Na$_V$Eh$^{\Delta N}$.

a. The flowchart of data process of Na$_V$Eh$^{\Delta N}$. Raw movies of Na$_V$Eh$^{\Delta N}$ were motion corrected, and the CTF values of the resulting micrographs were estimated by gctf, followed by 2D and 3D classifications. The best 3D class contained 81,844 particles, followed by per-particle polishing. And the polished particles were imported into CryoSPARC for non-uniform refinement with C1- and C4-symmetry, respectively. **b**. Local resolution distribution for Na$_V$Eh$^{\Delta N}$ map. **c**. Angular distribution of Na$_V$Eh$^{\Delta N}$ map. **d**. The FSC curves of the EM structure of Na$_V$Eh$^{\Delta N}$. **e**. EM densities for S1-S6 helices of Na$_V$Eh$^{\Delta N}$. **f**. EM densities for pore-loop of Na$_V$Eh^{WT_Ca} (green), Na$_V$Eh^{WT_EGTA} (pink), and Na$_V$Eh$^{\Delta N}$ (light purple).

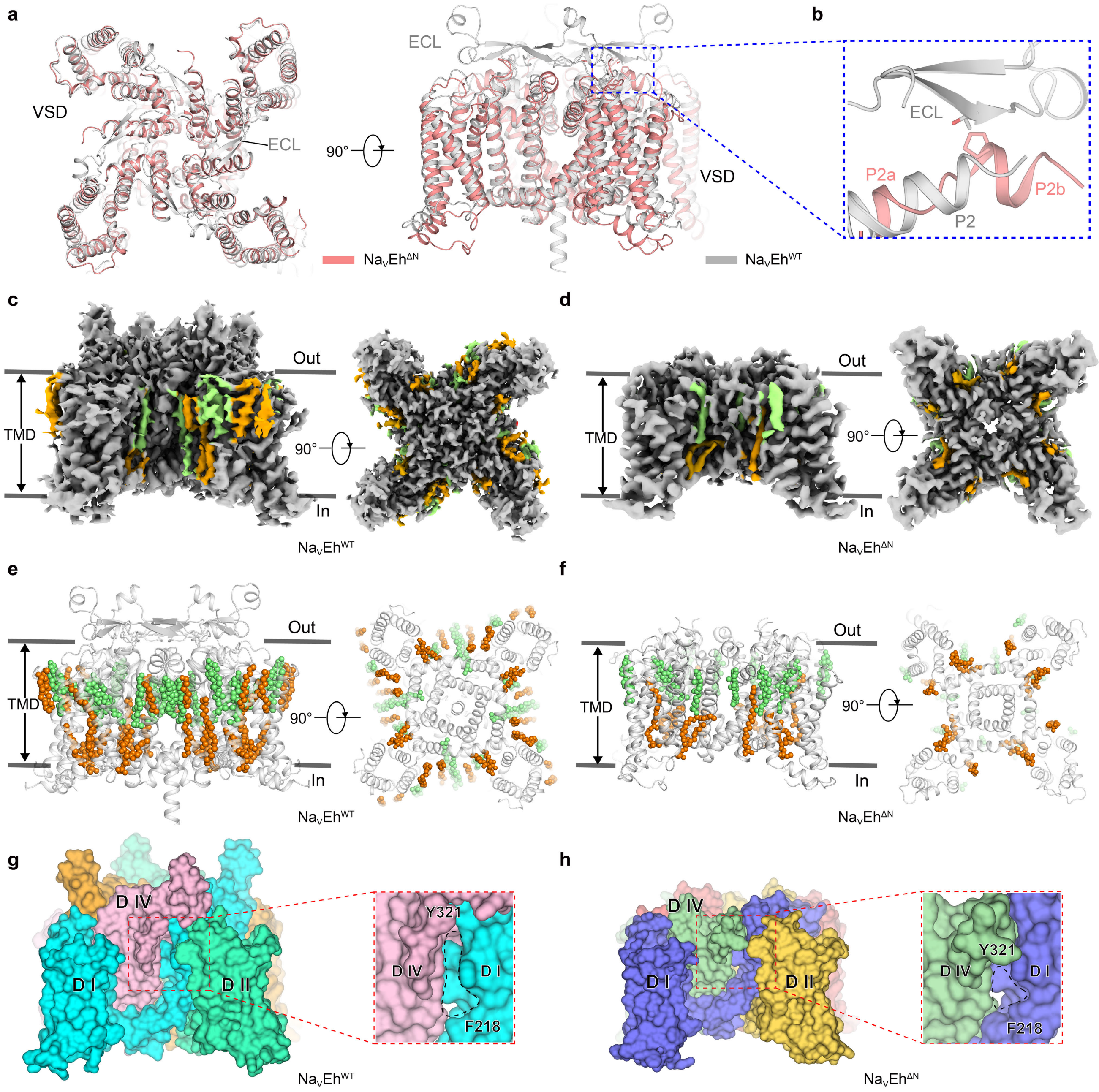

Supplementary Figure 6. Structural comparison of Na_VEh^{WT} and $Na_VEh^{\Delta N}$.

a. The superposition of $Na_VEh^{\Delta N}$ (red) and Na_VEh^{WT} (gray) are shown viewed from top-down (Left) and side (Right). **b.** Zoom-in view the ECL and P2 helix of $Na_VEh^{\Delta N}$ and Na_VEh^{WT}. **c** and **d.** The cryo-EM maps of Na_VEh^{WT} (**c**) and $Na_VEh^{\Delta N}$ (**d**). The protein, lipids, and cholesterol are colored in gray, yellow, and green, respectively. **e** and **f.** Cartoon representation of Na_VEh^{WT} (**c**) and $Na_VEh^{\Delta N}$ (**d**). **g** and **h.** The fenestration of Na_VEh^{WT} (**g**) and $Na_VEh^{\Delta N}$ (**h**). The fenestration between neighboring subunits is highlighted in red dashed squares.

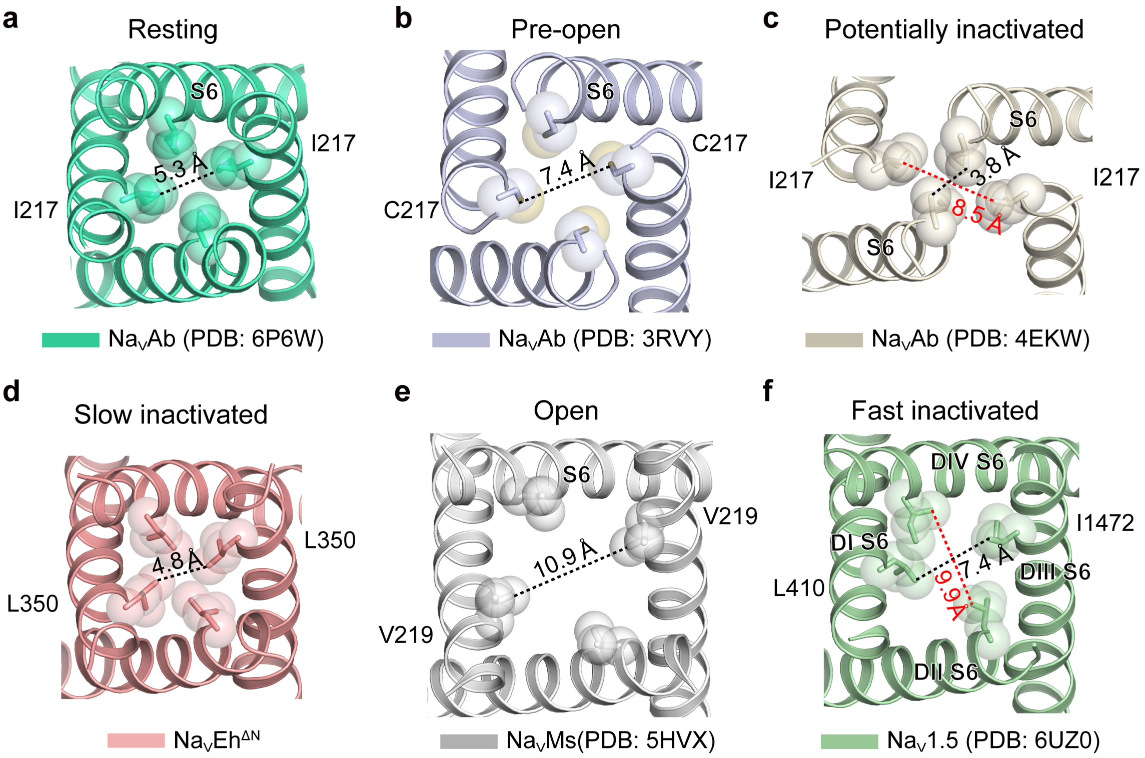

Supplementary Figure 7. The activation gates of Na_V channels

a. The activation gate of the resting-state Na_VAb^{WT} (PDB code: 6P6W, colored in green). **b.** The activation gate of the pre-open state Na_VAb^{I217C} (PDB code: 3RVY, colored in light blue). **c.** The activation gate of the inactivated Na_VAb^{WT} (PDB code: 4EKW, colored in wheat). **d.** The activation gate of the slow-inactivated $Na_VEh^{\Delta N}$. **e.** The activation gate of the open Na_VMs (PDB code: 5HVX, colored in gray). **f.** The activation gate of the fast-inactivated $Na_V1.5$ (PDB code: 6UZ0, colored in light green). The key hydrophobic residues are shown as sticks and spheres. The black and red dashed lines indicate the distances between opposing S6 helices of the activation gate.

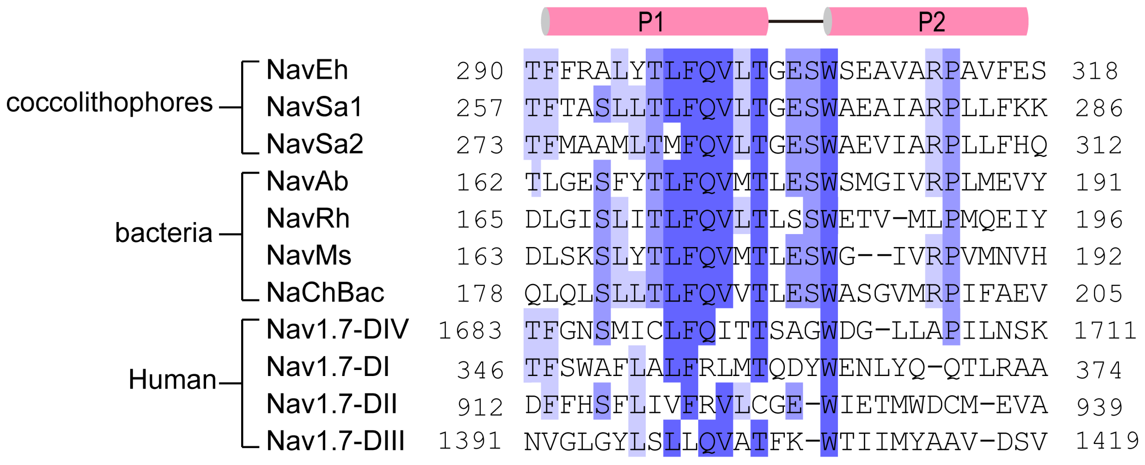

Supplementary Figure 8. Sequence alignment of the pore-loop Na_V channels.

Sequence alignment of the pore-loop of Na_VEh (Accession number CAMPEP_01 87645740), Na_VSa1 (Accession number CAMPEP_01 19314838), Na_VSa2 (Accession number CAMPEP_0119345692), Na_VAb (Uniprot: A8EVM5), Na_VRh (GI: 262276647), Na_VMs (Uniprot: A0L5S6), NaChBac (Uniprot: Q9KCR8) and human Na_V1.7(Uniprot: Q15858). Conserved residues are highlighted in blue. Cartoon representation of the P1 and P2 helices are labeled on the top of the sequence.

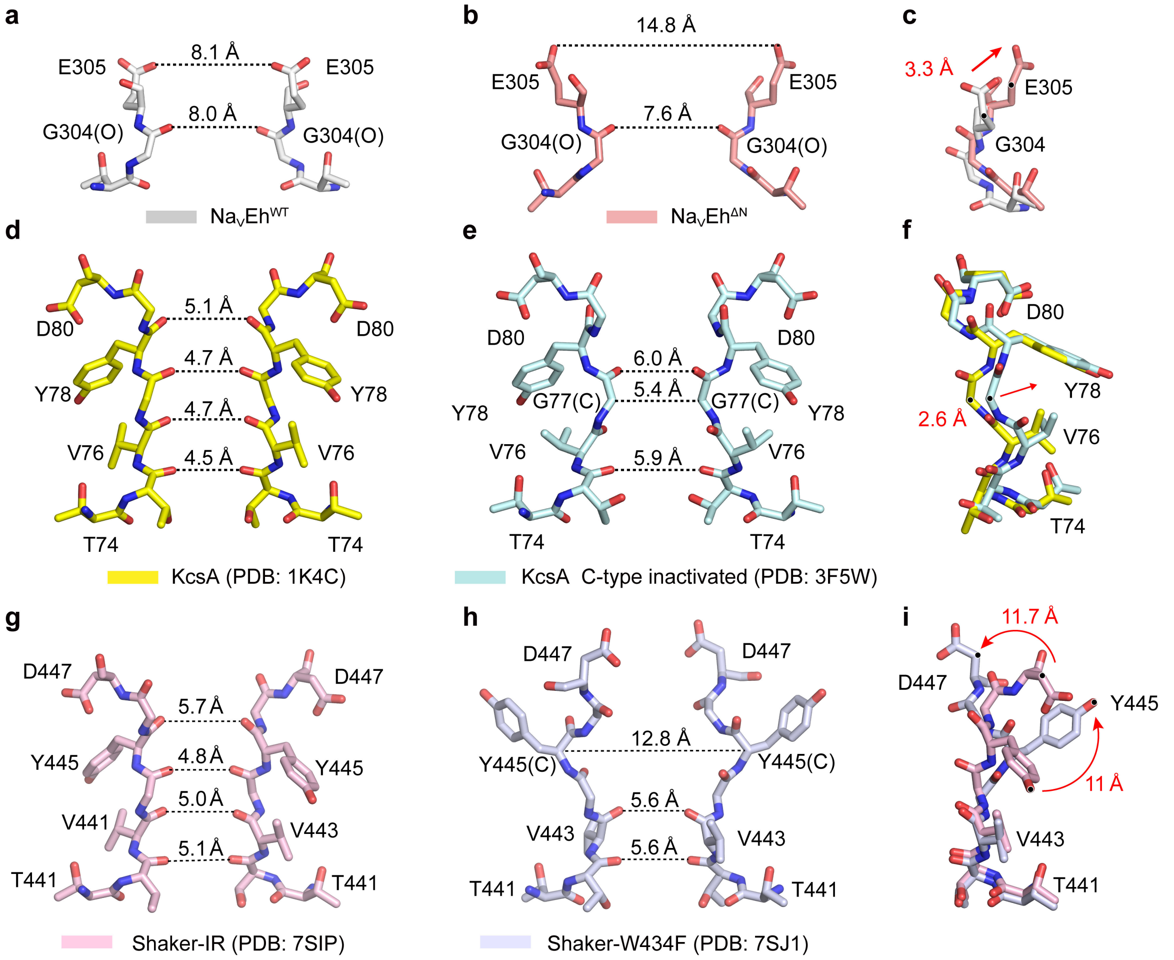

Supplementary Figure 9. Conformational changes in the SFs of Na$_V$ and potassium channels

a-b. The selectivity filter of Na$_V$Eh^{WT} (**a**) and Na$_V$Eh$^{\Delta N}$ (**b**). Black dashed lines represent the distances within the SFs. The letters in parentheses represent backbone atoms, O for carbonyl oxygen. **c.** The superposition of the SFs of Na$_V$Eh^{WT} (gray) and Na$_V$Eh$^{\Delta N}$ (red). The red arrow indicates the shifts of E305 between the two SFs. **d-e.** The selectivity filter of KcsA (PDB code: 1K4C, colored in yellow) and KcsA in C-type inactivated state (PDB code: 3F5W, colored in cyan). The letters in parentheses stands for backbone group, C for Cα atoms. **f.** The superposition of the SFs of KcsA (yellow) and KcsA in C-type inactivated state (cyan). The red arrow indicates the shifts of G77 between the two SFs. **g-h.** The selectivity filter of Shaker-IR (PDB code: 7SIP, colored in pink) and Shaker-W434F (PDB code: 7SJ1, colored in lightblue). Black dashed lines represent the distances within the SFs. The letters in parentheses stand for backbone group, C for Cα atoms. **i.** The superposition of the SF of Shaker-IR (pink) and Shaker-W434F (light blue). The red arrow indicates the shifts of Y455 and D447 between the two SFs.

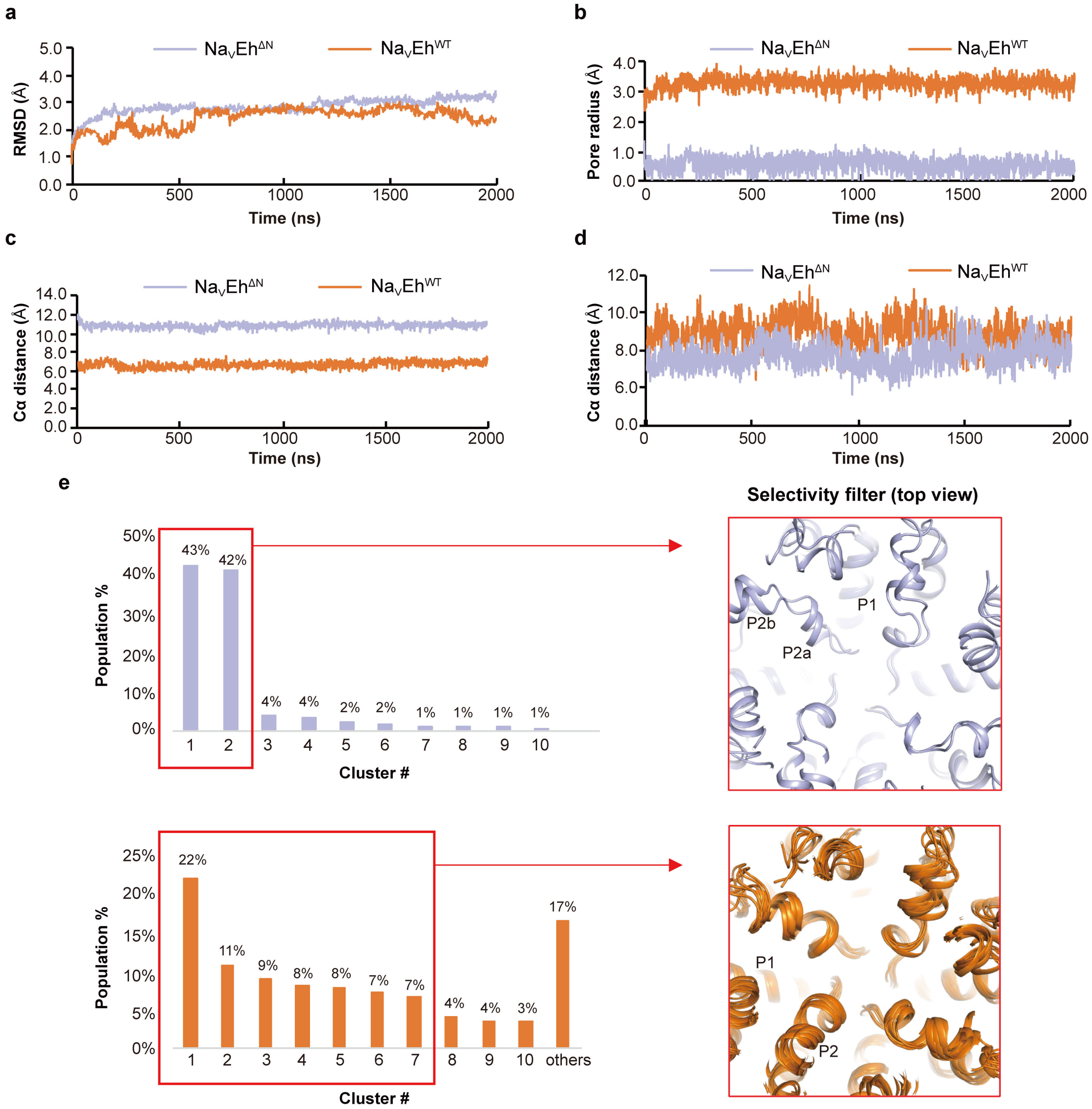

Supplementary Figure 10. MD simulations of the pore domain of Na_VEh^{WT} and $Na_VEh^{\Delta N}$.

a. The protein backbone RMSD plots for each simulation system. The protein structures in each trajectory were aligned with the initial structure using the Least Squares algorithm before computing the RMSD. **b.** Dynamics of pore radius. For Na_VEh^{WT}, the N helix was included during the simulation, but it was removed for the calculation of the pore radius using the HOLE program. **c.** Dynamics of distance between Ca atoms of A310 and P314. This distance was utilized as an indicator of the dilation of the selectivity filter. **d.** Dynamics of distance between side centroids of F299 and F218. This distance served as a measure of the fenestration in the protein structure. In panel c and d, when the distance of interest involved four values due to the symmetry of the protein, the minimum of the four values was used for the plot. **e.** Cluster analysis of 2000 ns MD simulation for each system. The major clusters accounting for over 5% of the population were indicated with red boxes and the conformations of the selectivity filter from representative structures from these clusters were displayed.

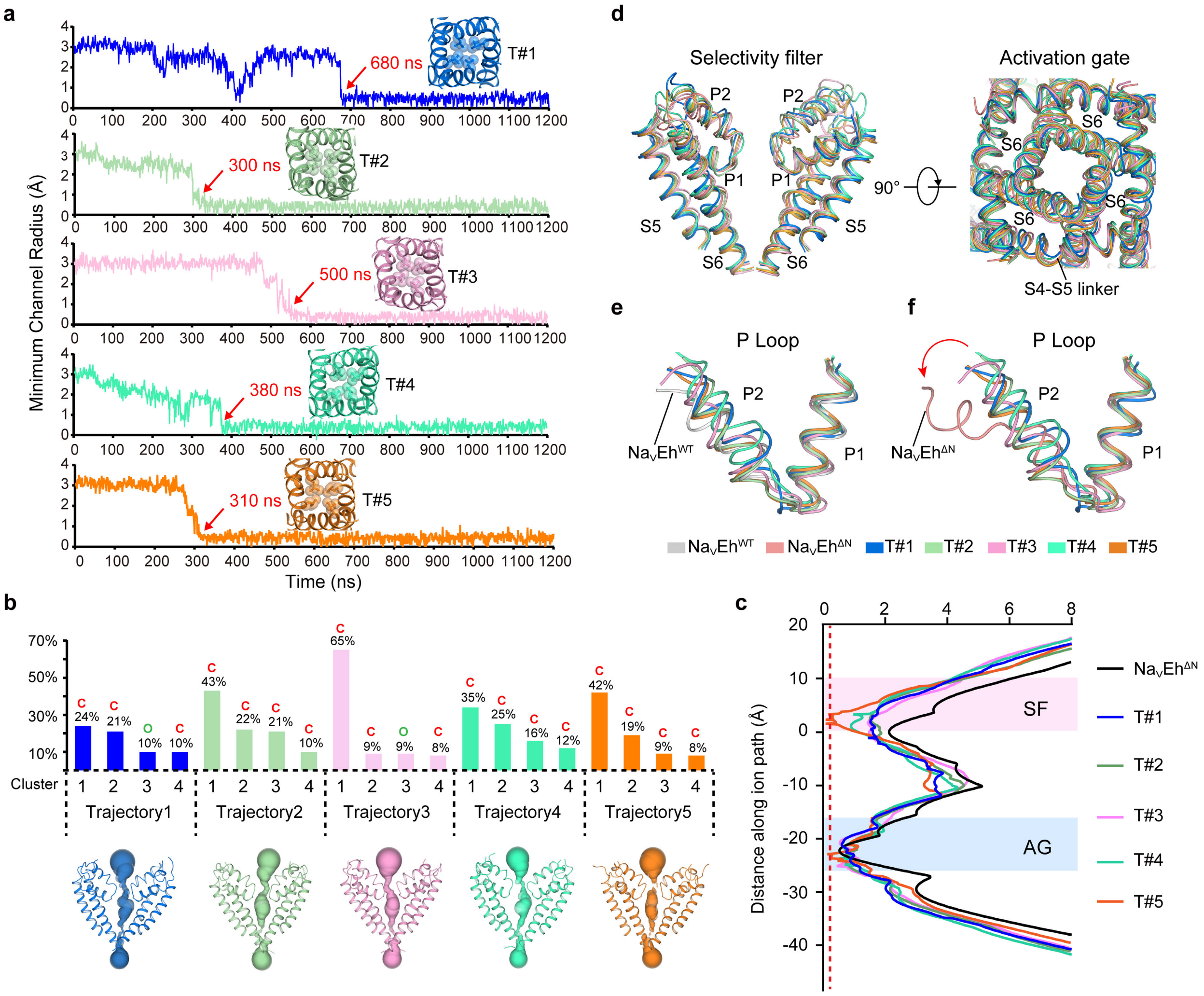

Supplementary Figure 11. MD simulations of Na$_V$Eh pore domain without the N-helix.

a. Pore radius fluctuations in the five independent MD simulation trajectories of Na$_V$Eh pore domain without the N-helix. Five independent production runs were performed. T represents Trajectory, and the time point at which the gate closed is indicated with a red arrow. The structures of closed activation gate from MD are displayed as cartoons. The visualized duration here is limited to 1,200 ns. **b**. Clustering analysis on full-length trajectories of the Na$_V$Eh pore domain without the N-helix. The ion path of the representative structure from the most populated cluster is depicted for each trajectory. In the bar chart, the closed gate is represented by a red "C" and the open gate is represented by a green "O". **c**. The pore radii of the representative structures from the most populated cluster of each trajectory for Na$_V$Eh pore domain without the N-helix. **d**. The superposition of the five representative structures from the most populated cluster of each trajectory and the Na$_V$Eh$^{\Delta N}$ pore (red). **e** and **f**. Pore-loop comparison between and the five representative structures of the MD simulations from panel d with Na$_V$Eh^{WT} (e) and Na$_V$Eh$^{\Delta N}$ (f). Red arrow indicates the conformational difference between the P2 helices.

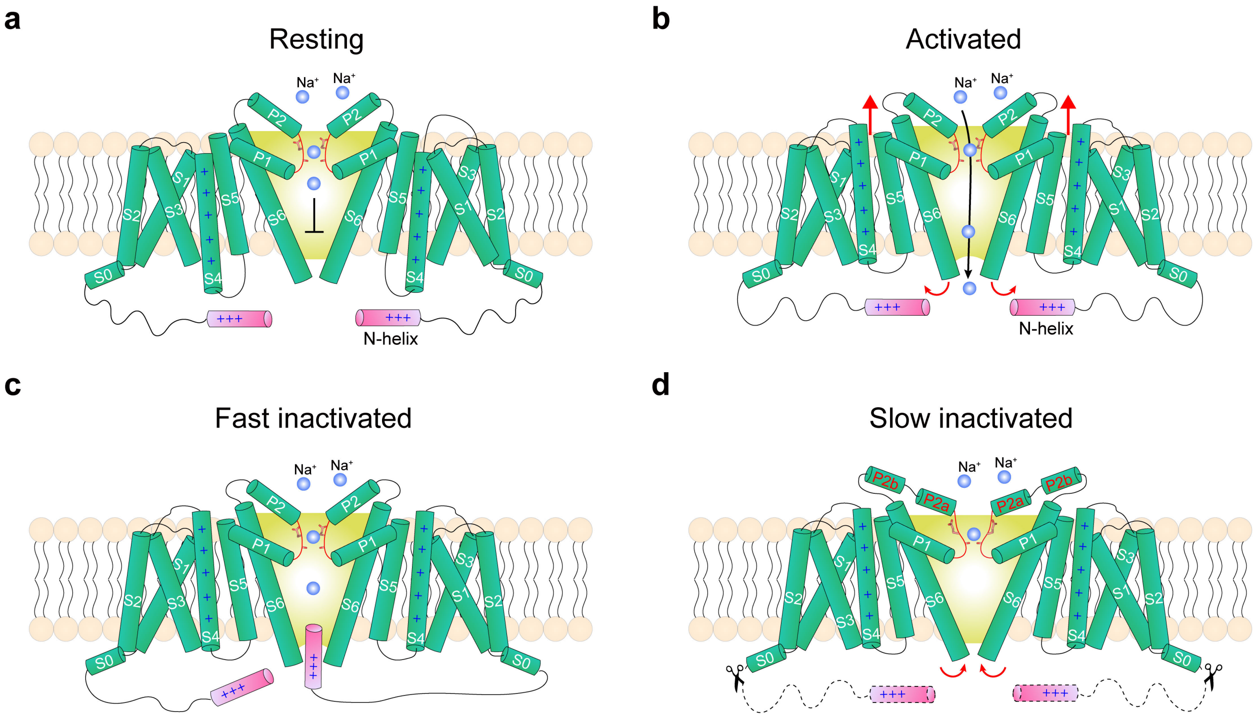

Supplementary Figure 12. Proposed inactivation mechanisms of NavEh.

a. Resting state of Na_vEh in the hyperpolarized membrane potential. The voltage-sensors assume "down" conformation, and the inner activation gate is closed. **b.** Activated open state of Na_vEh. The voltage sensor activation is accompanied by the upward movements of S4, and followed by gate opening, then the channel is conductive. **c.** Fast inactivated state of Na_vEh. Several milliseconds after the gate opening, the N-helix plugs into the open activation gate and blocks it, resulting in the fast-inactivated state. **d.** Slow inactivated state of Na_vEh. When the N-helix was deleted, with prolonged depolarizing stimuli, the selectivity filter and the activation gate underwent conformational changes, resulting in the slow inactivated state. Purple spheres represent Na^+ ions. Red arrows indicate conformational shifts.

Supplementary Table 1. Cryo-EM data collection, refinement, and validation statistics

	$Na_VEh^{\Delta N}$ (EMDB-36042) (PDB: 8J7M)	$Na_VEh^{WT\_Ca}$ (EMDB-36039) (PDB: 8J7F)	$Na_VEh^{WT\_EGTA}$ (EMDB-36041) (PDB: 8J7H)
**Data collection and processing**			
Magnification	130,000 ×	130,000 ×	130,000 ×
Voltage (kV)	300	300	300
Electron exposure (e^-/Å^2)	60	60	60
Defocus range (μm)	−1.2 ~ −2.2	−1.2 ~ −2.2	−1.2 ~ −2.2
Pixel size (Å)	1.04	1.04	1.04
Symmetry imposed	C4	C4	C4
Initial particle images (no.)	454,312	447,915	352,310
Final particle images (no.)	81,844	110,665	59,377
Map resolution (Å)	3.10	2.60	3.30
FSC threshold	0.143	0.143	0.143
Map resolution range (Å)	2.5 ~ 4.5	2.2 ~ 4.6	2.6 ~ 5.0
**Refinement**			
Initial model used (PDB code)	7X5V	7X5V	7X5V
Model resolution (Å)	3.40	2.80	3.40
FSC threshold	0.5	0.5	0.5
Map sharpening *B* factor (Å^2)	-110.1	-68.7	-120.1
Model composition			
Non-hydrogen atoms	8,976	10,436	9,457
Protein residues	1,028	1,176	1,180
Ligands	20	53	0
B factors (Å^2)			
Protein	39.03	38.52	34.61
Ligand	26.14	36.91	
R.m.s. deviations			
Bond lengths (Å)	0.010	0.008	0.004
Bond angles (°)	1.260	1.300	0.746
Validation			
MolProbity score	2.13	2.08	1.93
Clashscore	12.00	19.00	13.00
Poor rotamer (%)	0.0	1.4	0.0
Ramachandran plot			
Favored (%)	91.50	95.80	95.56
Allowed (%)	8.50	4.12	4.44
Disallowed (%)	0.00	0.09	0.00

Supplementary Table 2. Composition of electrophysiology solutions (mM).

HEPES, 4-(2-hydroxyethyl)-1-piperazineethanesulfonic acid; E: Extracellular solution; I: Intracellular solution.

	E_Ca^{2+}	I_0 Ca^{2+}	E_0 Ca^{2+}	E_EGTA	I_Ca^{2+}
NaCl	140	10	140	140	10
KCl	4	-	4	4	-
$MgCl_2$	1	-	1	1	-
$CaCl_2$	1	-	-	-	1
CsF	-	140	-	-	140
HEPES	10	10	10	10	10
EGTA	-	1	-	1	-
Glucose	10	-	10	10	-
pH	7.3	7.3	7.3	7.3	7.3
	NaOH	CsOH	NaOH	NaOH	CsOH
